# Structure of the Head of the *Bartonella* Adhesin BadA

**DOI:** 10.1371/journal.ppat.1000119

**Published:** 2008-08-08

**Authors:** Pawel Szczesny, Dirk Linke, Astrid Ursinus, Kerstin Bär, Heinz Schwarz, Tanja M. Riess, Volkhard A. J. Kempf, Andrei N. Lupas, Jörg Martin, Kornelius Zeth

**Affiliations:** 1 Department of Protein Evolution, Max Planck Institute for Developmental Biology, Tübingen, Germany; 2 Department of Bioinformatics, Institute of Biochemistry and Biophysics, Polish Academy of Sciences, Warsaw, Poland; 3 Institut für Medizinische Mikrobiologie und Hygiene, Eberhard-Karls-Universität, Tübingen, Germany; University of California San Diego, United States of America

## Abstract

Trimeric autotransporter adhesins (TAAs) are a major class of proteins by which pathogenic proteobacteria adhere to their hosts. Prominent examples include *Yersinia* YadA, *Haemophilus* Hia and Hsf, *Moraxella* UspA1 and A2, and *Neisseria* NadA. TAAs also occur in symbiotic and environmental species and presumably represent a general solution to the problem of adhesion in proteobacteria. The general structure of TAAs follows a head-stalk-anchor architecture, where the heads are the primary mediators of attachment and autoagglutination. In the major adhesin of *Bartonella henselae*, BadA, the head consists of three domains, the N-terminal of which shows strong sequence similarity to the head of *Yersinia* YadA. The two other domains were not recognizably similar to any protein of known structure. We therefore determined their crystal structure to a resolution of 1.1 Å. Both domains are β-prisms, the N-terminal one formed by interleaved, five-stranded β-meanders parallel to the trimer axis and the C-terminal one by five-stranded β-meanders orthogonal to the axis. Despite the absence of statistically significant sequence similarity, the two domains are structurally similar to domains from *Haemophilus* Hia, albeit in permuted order. Thus, the BadA head appears to be a chimera of domains seen in two other TAAs, YadA and Hia, highlighting the combinatorial evolutionary strategy taken by pathogens.

## Author Summary

The ability to adhere is an important aspect of the interaction between bacteria and their environment. Adhesion allows them to aggregate into colonies, form biofilms with other species, and colonize surfaces. Where the surfaces are provided by other organisms, adhesion can lead to a wide range of outcomes, from symbiosis to pathogenicity. In Proteobacteria, colonization of the host depends on a wide range of adhesive surface molecules, among which Trimeric Autotransporter Adhesins (TAAs) represent a major class. In electron micrographs, TAAs resemble lollipops projecting from the bacterial surface, and in all investigated cases, the adhesive properties reside in their heads. We have determined the head structure of BadA, the major adhesin of *Bartonella henselae*. This pathogen causes cat scratch disease in humans, but can lead to much more severe disease in immunosuppressed patients, e.g., during chemotherapy or after HIV infection. Surprisingly, domains previously seen in other TAA heads are combined in a novel assembly, illustrating how pathogens rearrange available building blocks to create new adhesive surface molecules.

## Introduction

Adherence to the host is a key event in bacterial pathogenesis. The mediators of this process, called adhesins, form a heterogenous group that vary in architecture, domain content and mechanism of binding. Trimeric autotransporter adhesins, also referred to as OCAs for oligomeric coiled-coil adhesins, form a new class of adhesins recently defined from pathogenic proteobacteria [1]–[4]. The best studied TAAs are important virulence factors: YadA of *Yersinia enterocolitica*, a species causing enteritis, mesenteric lymphadenitis, and reactive arthritis in humans [5],[6]; NadA of *Neisseria meningitidis* [7], an agent of meningitis and sepsis; UspA1 and A2 of *Moraxella catarrhalis* [4],[8], a prominent species in respiratory tract infections; Hia and Hsf of *Haemophilus influenzae* [9],[10], an organism causing meningitis and respiratory tract infections, and BadA of *Bartonella henselae* [11], which is the agent of cat scratch disease. In the context of the AIDS pandemic, *Bartonella henselae* has also emerged as the agent of bacillary angiomatosis, an uncontrolled proliferation of blood vessels resulting in tumor-like masses of cells in patients with impaired immune systems.

All TAAs follow a head-stalk-anchor architecture in the direction from amino- to carboxy-terminus of the protein [4]. Whereas head and stalk are assembled from an array of analogous domains, the anchor is homologous in all TAAs and represents the defining element of this family [3]. It trimerizes in the outer membrane to form a 12-stranded pore [12], through which the head and the stalk exit the cell. After export is completed, the C-terminal end of the folded stalk occludes the pore. The head, which is projected above the cell surface by the stalk, mediates a range of molecular interactions such as autoagglutination and attachment to host tissue, typically via proteins of the extracellular matrix, e.g. collagen, fibronectin, or laminin. Two head structures, a complete one from YadA [13] and a partial one from Hia [14], have been solved by X-ray crystallography, revealing fundamentally different trimeric complexes with novel folds.

**Figure 1:**
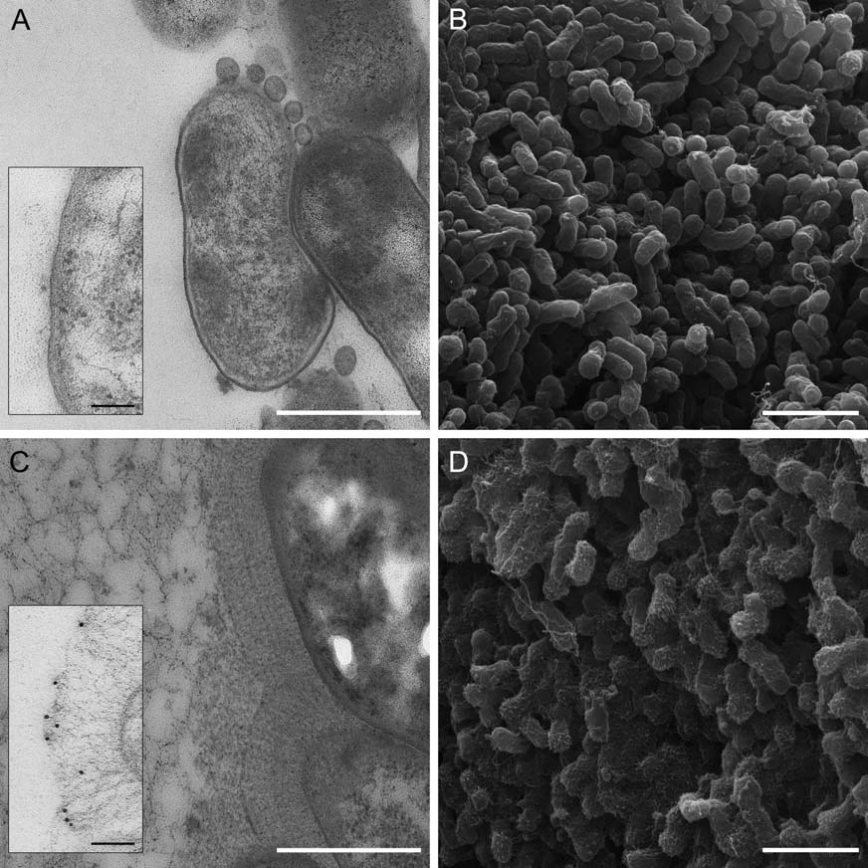
EM pictures of BadA. Transmission (left) and scanning electron micrographs (right) of *Bartonella henselae*. Panels A and B show a mutant strain deficient in BadA, panels C and D show the wildtype bacteria grown on blood agar plates. The scale bars are 500 nm in the TEM pictures (A and C) and 2 µm in the SEM pictures (B and D). BadA has a length of 240+/−10 nm. The inserts in panels A and C (scale bar 100 nm) show bacteria after on-section labeling with an antibody raised against the C-terminal head fragment of BadA. The head structure is found exclusively at the tips of the elongated fibers in panel C.

Of the experimentally studied TAAs, *Bartonella henselae* BadA is the longest representative, at over 3000 residues, and extends approx. 240 nm from the bacterial cell surface (Figure 1). BadA has been shown to bind collagen and fibronectin [11]; although the location of the binding sites has not been determined, comparison to other TAAs suggests that they reside in the head. The BadA head is composed of two parts, the N-terminal of which is clearly homologous to the head of YadA, while the C-terminal has no detectable similarity to proteins of known structure. We have recently produced a comprehensive, web-based annotation platform for TAAs [15]; as part of this work, we found that the C-terminal part of the BadA head in fact consists of two domains. Here we report the crystal structure of these two domains, which closely resemble parts of the Hia head structure despite an extremely low sequence similarity. Based on our data and a homology model to the YadA head, we present the structure of the full BadA head.

## Results/Discussion

### Sequence analysis

**Figure 2:**
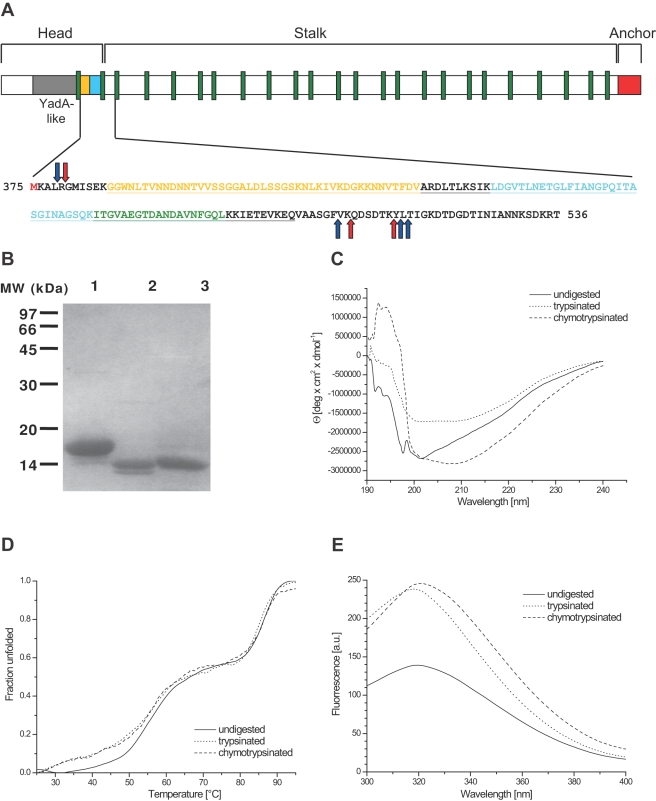
Domain structure of BadA and sequence of the fragment used in this study. (A) The domain arrangement of BadA, with the YadA-like head in grey, the two domains described in this paper in gold and cyan, respectively, the neck sequences in green and the membrane anchor in red. The lower panel shows the sequence of the fragment used in this study, colored according to the domain arrangement. Red (trypsin) and blue (chymotrypsin) arrows indicate protease cleavage sites; several variants of a protease-resistant 14 kDa core fragment were found by mass spectrometry. Underlined is the part of the sequence that is resolved in the crystal structure, which correlates well with the protease-resistant part of the protein. (B) SDS-PAGE of the 17 kDa fragment before (lane 1) and after trypsin (lane 2) or chymotrypsin (lane 3) treatment. (C) CD spectra of the fragments before and after proteolysis. A higher fraction of random coil signal contributes to the spectrum of the undigested fragment. (D) Heat denaturation of the fragments before and after proteolysis measured at 210 nm wavelength. Unfolding occurs in two steps. (E) Fluorescence spectra of the fragments before and after proteolysis.

With a size of 3082 residues per monomer, BadA (gi|119890727|) is considerably larger than other well-studied TAAs, such as YadA (455 res.), Hia (1098 res.), UspA1 (863 res.) or NadA (364 res.). Although very long (240 nm+/−10 nm, Figure 1), it preserves the head-stalk-anchor architecture typical of TAAs. The sequence is highly repetitive and the presence of 24 conserved connectors, called neck sequences [4], allowed us to define domain boundaries and to parse out the head, stalk and anchor regions [11]. We found that the head region falls into two parts, separated by a neck sequence (Figure 2A). The first part was evidently similar to the head of YadA (Protein Data Bank (PDB) code 1p9h), as well as to the heads of many other TAAs of unknown structure, due to the periodic occurrence of degenerate SVAIG motifs, which form the inner β-strands of the trimeric β-helix [13]. The second part however showed no discernible similarity to any protein of known structure, even when using advanced sequence comparison and fold prediction tools (see Methods). By sequence comparisons to other TAAs, we determined that this part in fact consists of two separate domains, one containing a highly conserved Gly-Trp (GW) motif near its N-terminus and the other a conspicuous Gly-Ile-Asn (GIN) motif near its C-terminus [15]; we therefore named the former “Trp-ring domain“ and the latter “GIN domain”. Based on this analysis, we decided to determine the structure of these two domains. The fragment chosen for crystallization extended from the end of the YadA-like head domain to the end of the first stalk segment (residues 375 to 536, Figure 2A).

### Determination of trimer stability and proteolytic processing

The recombinantly expressed BadA construct runs as a trimer of three 17 kDa subunits on calibrated size exclusion columns (data not shown). To assay the stability of the trimers, we subjected the protein to proteolytic treatment with trypsin and chymotrypsin. In both cases we obtained fragments of approx. 14 kDa (Figure 2B, Figure S1), which could still form trimers. Mass spectrometric analysis showed that the carboxy-terminal part of the construct was particularly prone to digestion, suggesting a flexible conformation. The termini of the protease-resistant fragment are marked by red arrows in Figure 2A.

The CD spectra of the digested and undigested forms indicated well-folded proteins consisting primarily of β-sheets (Figure 2C). Thermal denaturation curves with CD detection showed that unfolding was also very similar in these proteins and proceeded cooperatively as a two-step process (Figure 2D), with a first plateau at 75°C and complete unfolding at 92°C. This elevated stability seems common to TAA domains, as shown for the anchor of YadA [16] and for the complete YadA protein [17]. The two-step denaturation may reflect the presence of two domains in our construct.

The single tryptophan residue close to the amino-terminus (Trp387) allowed us to perform fluorescence measurements. The λ_max_ of 320 nm, which is typical for buried Trp residues in folded proteins, does not change significantly in the trypsinized and chymotrypsinized fragments, but the intensity of the emission signal increases substantially (Figure 2E). Proteolysis of the amino-terminal sequence may have removed quenching residues from the vicinity of the tryptophans.

### Crystallization

The undigested protein was crystallized under a variety of conditions. The crystals typically grew to 300×200 µm in size, were well ordered, and diffracted up to a resolution of 1.1 Å. All crystals tested, although of different shape and from different crystallization conditions, belonged to space group P1 with cell constants of a = 29.87, b = 51.14, c = 58.62, α = 65.87°, β = 76.6°, γ = 82.08°. In order to solve the structure of this fragment, a variety of heavy atom derivatives were prepared and data were collected, but none of the crystals showed binding. This was not unexpected, as the protein does not contain cysteine or methionine residues, which commonly bind heavy metal compounds. For the same reason, we could not use selenomethionine-based MAD-phasing.

### Homology Modeling and Structure Determination

The continued failure to determine the structure experimentally led us to re-explore the protein with bioinformatic tools. We had failed to identify potential homologs through either sequence comparisons or fold recognition, but a new method for detecting distant sequence similarity by comparing profile Hidden Markov Models with each other had just been developed in our department (see Methods). The two best matches obtained with this method, albeit with low statistical confidence, were to the structure of *Haemophilus* Hia (PDB code 1s7m). The two BadA domains each gave a separate match – the first domain to the C-terminal part of Hia and the second to the N-terminal part. These matches were intriguing, as Hia is also a TAA and the conserved GW and GIN motifs, which we had identified as key signatures of these domains [15], were also present in the domains from Hia. The inverted order of the two domains in the Hia structure relative to BadA provides a rationale for the inability of less sensitive methods to detect the relationship between the two proteins.

We therefore attempted to solve the BadA structure by molecular replacement with homology models based on the Hia structure. To this end, we built full-atom models for each domain, as described in the Methods section. Molecular replacement searches returned two solutions, which were further refined, and a trimeric model of residues 385 to 498 of BadA could be built into the electron density map (underlined in Figure 2A). The statistics given in Table 1 demonstrate the high overall quality of this structure. The termini of the construct were not resolved, as expected from the results of proteolytic digestion, which suggested that they are unstructured.

**Table 1:** Summary of data collection and refinement statistics.

Data collection^1^	
Wavelength [Å]	0.9787
Space group	P1
Cell constants [Å/degree]	29.87, 51.14, 58.62/65.87, 76.60, 82.08
Resolution [Å]	20.0–1.13 (1.20–1.13)
Unique reflections	105179 (14273)
Redundancy	5.8 (5.4)
Completeness [%]	90.7 (76.0)
R_merge_ [%]	8.5 (52.4)
I/σ(I)	12.7 (3.7)
Wilson B-factor	13.6
**Refinement statistics** 1	
Space group	P1
Resolution [Å]	20.0–1.13 (1.20–1.13)
R_cryst_	0.15 (0.22)
R_free_	0.17 (0.23)
Non-hydrogen atoms	2870
Waters	438
Mean B-value (Å^2^)	10.8
r.m.s.d. of bond length (Å^2^)	0.01
r.m.s.d. of angle (deg)	1.3
**Model quality**	
Residues in most favored region	328 (98.5%)
Residues in most allowed region	4 (1.2%)
Residues in outlier region	1 (0.3%)
Residues in alternate conformations	23 (6.9%)

1 Numbers in parenthesis refer to the highest resolution shell.

### Structure of the two BadA head domains at atomic resolution

**Figure 3:**
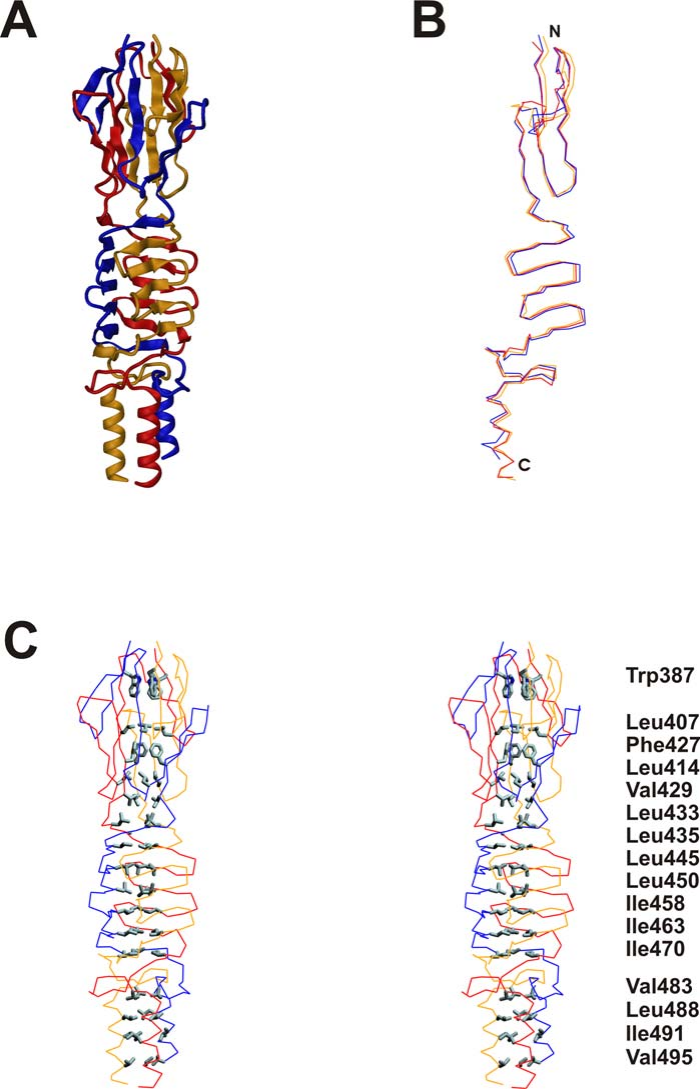
Quartenary structure of the BadA head domain. (A) Structure of the entire BadA head fragment. The three independent protein chains are colored in yellow, red and blue. (B) Superposition of the three individual protein chains. A significant deviation is visible in particular at the N-terminal part of the structure. (C) Stereo representation of the hydrophobic core of the protein, which is built by 16 residues related by threefold symmetry.

The overall structure of the construct is rod-like, with a length of 10 nm and an approximate diameter of 2.5 nm. Superposition of the three protein chains shows root mean square deviations (r.m.s.d.) of ∼1.2 Å, with the main differences in the termini and in the loops. Although the structural variability near the termini is probably an artifact of expressing a truncated construct, the overall r.m.s.d suggest an intrinsic flexibility of the three protein chains while the B-factors are low and equally distributed all over the protein chain except for the coiled-coil part. The three chains are tightly intertwined and each can only assume its structure in the context of the other two. 72 of 114 residues from each chain (63%) are involved in intersubunit contacts, including 16 residues in the hydrophobic core of the trimer (Figure 3). More than 50 hydrogen bonds (as defined by a cutoff distance of 3.5 Å) are formed between any two chains. There are, however, no inter-subunit salt bridges and only two intra-subunit ones (Asp394 - Lys418, and Asp 481 - Lys469). Overall, 5070 Å^2^ from each chain, corresponding to half of its total surface area of 10040 Å^2^, are buried in the trimer.

**Figure 4:**
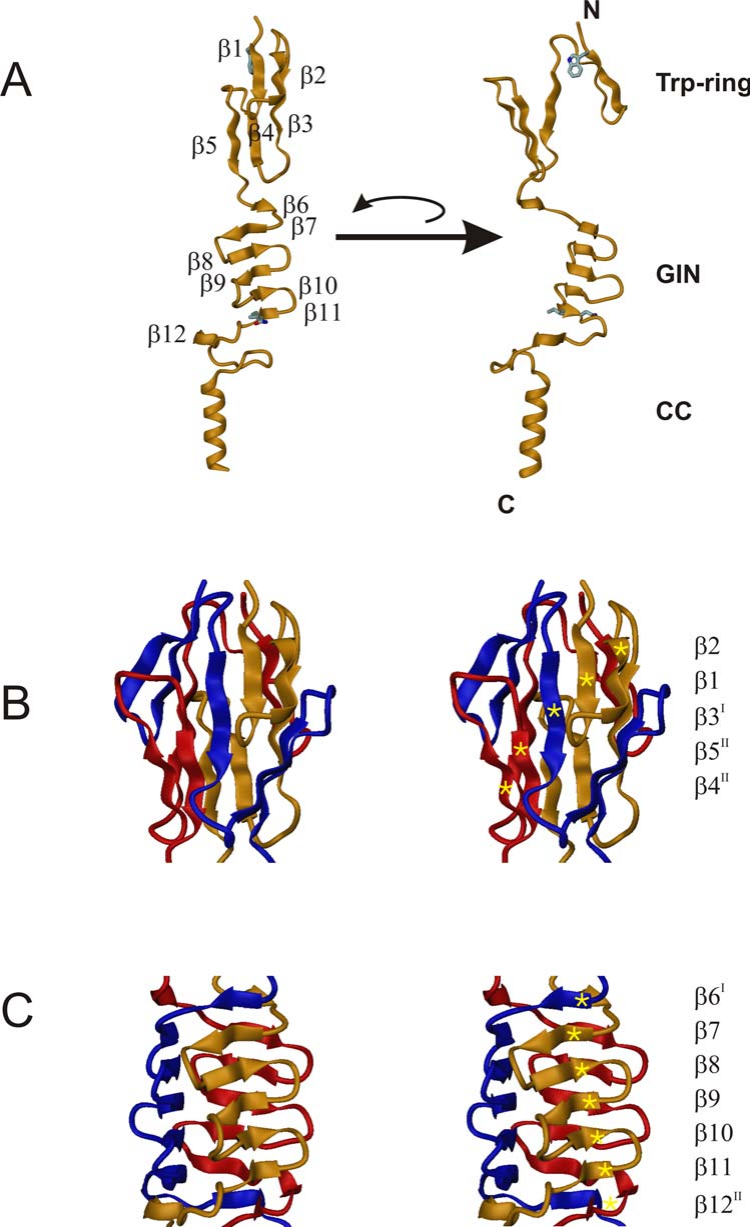
Crystal structure of the BadA head domain. (A) Structure of the monomeric BadA fragment in ribbon representation with the secondary structure elements marked (β1-β12). Two individual orientations rotated around the threefold axis by 90 degrees relative to each other are shown, and the two domains (Trp-ring domain and GIN domain) and the coiled-coil part (CC) are indicated. The conserved residues Trp387 of the Trp-ring and Gly462-Ile463-Asn464 of the GIN domain which determine their nomenclature are shown in stick representation. (B) Structure of the N-terminal Trp-ring domain in stereo representation. The three independent protein chains are color-coded in yellow, red and blue. The three chains form intertwined mixed parallel/anti-parallel β-sheets, one of which is labeled with gold stars. Its β-strand sequence is β2-β1-β3^I^-β5^II^-β4^II^. (C) Structure of the GIN domain in stereo representation, with the individual monomers color-coded in yellow, red and blue. The three interdigitated chains form three β-sheets, one of which is labeled with gold stars. Its β-strand sequence is β6^I^-β7-β8-β9-β10-β11-β12^II^. The strand progression of the individual sheets is anti-parallel (β7-β11) while interacting strands from adjacent chains are combined via parallel strand pairing (β6^I^-β7 and β11-β12^II^).

**Figure 5:**
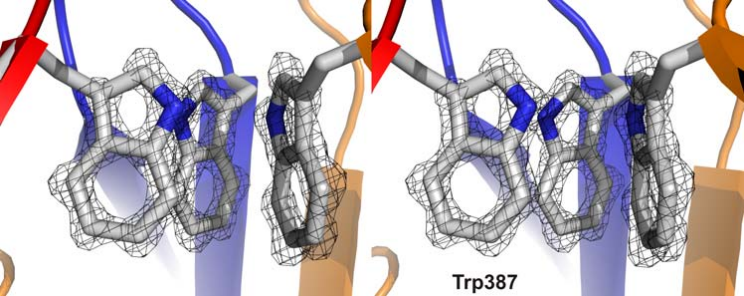
Crystal structure of the BadA head domain - omit map. The |F_obs_-F_calc_| electron density around the three Trp387 residues of the Trp-ring domain (calculated after simulated annealing with the Trp sidechains omitted), contoured at 3.5 sigma level.

The structure consists of four distinct elements, as anticipated from the sequence analysis (Figures 2A, 3 and 4): the Trp-ring domain, named for the peculiar arrangement of the highly conserved Trp residues (Figure 5), the GIN domain, a neck sequence, and a short segment of the coiled-coil stalk of BadA.

The Trp-ring domain forms a β-prism of interleaved, five-stranded β-meanders parallel to the trimer axis. Each of the three β-sheets forming the sides of the prism consists of the β1- β2 hairpin of one chain, β3^I^ of the next chain and the β4^II^- β5^II^ hairpin of the last chain, as viewed clockwise from the N-terminus; the strand order is β2- β1- β3^I^- β5^II^- β4^II^ (Figure 4B).

The GIN domain (residues 435–466) also forms a β-prism of 5-stranded β-meanders, albeit not interleaved and perpendicular to the trimer axis. Its five β-strands (β7- β11) are extended N-terminally by the region connecting GIN with the Trp-ring domain in the next chain (β6)^I^ and C-terminally by the first residues of the neck sequence in the last chain (β12^II^), again as viewed clockwise from the N-terminus (Figure 4C).

**Figure 6:**
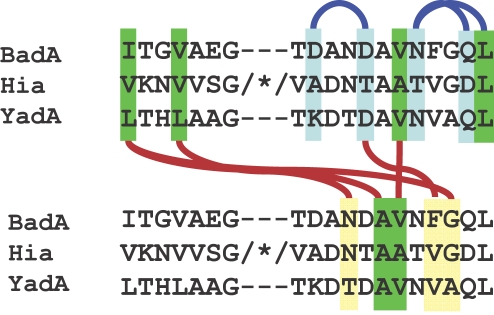
Network of hydrogen bonds in the necks of BadA, YadA and Hia. Intramolecular H-bonds are marked as blue lines, intermolecular ones as red lines. Residues involved in intermolecular H-bonds are yellow, residues involved in intramolecular H-bonds are blue, highly conserved hydrophobic core residues that also contribute to the H-bond network are green. Note that all H-bonds involve atoms of the main chains, which explains the low conservation of sidechains between neck sequences. * marks the insertion in the Hia sequence (see text).

The neck sequence serves as a connector, which makes the transition from the wide diameter of the β-prisms to the narrower diameter of the coiled-coil stalk. Although being largely devoid of regular secondary structure, the neck forms an extended network of hydrogen bonds (Figure 6). The coiled coil following the neck is a part of the extended stalk domain of BadA [11]. In our construct, we had included 50 residues from the N-terminal part of the BadA stalk, but only two heptads are visible in the structure, the rest being disordered.

### Structure comparison to other TAA domains

**Figure 7:**
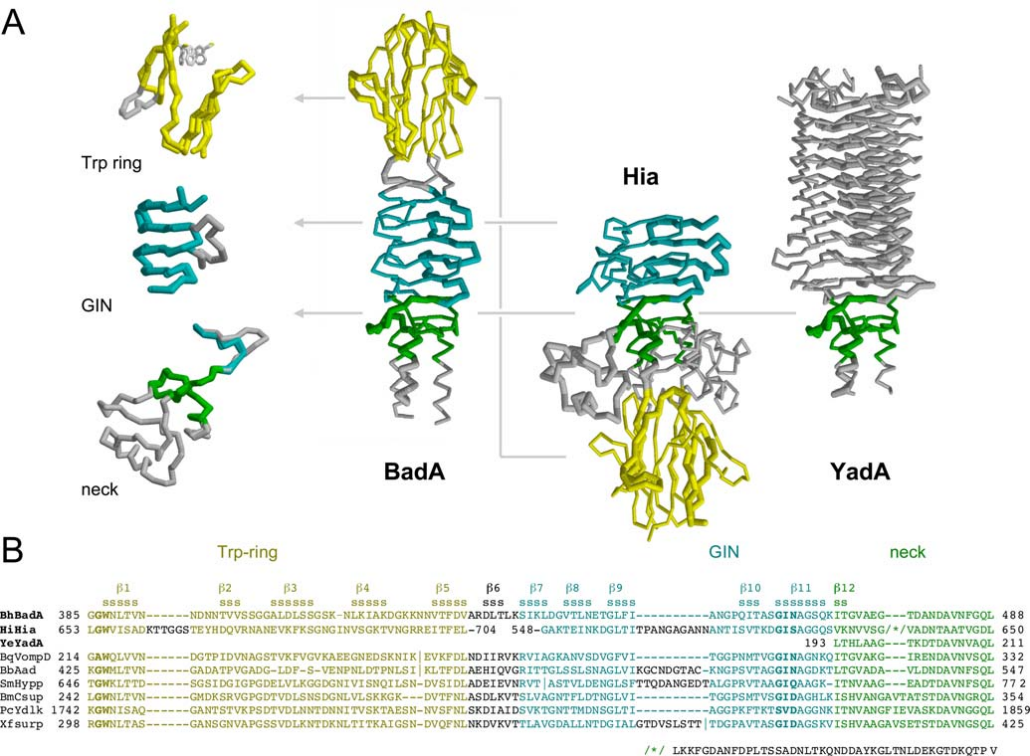
Structure comparison of the complete BadA head with YadA from *Y. enterolitica* and Hia from *H. influenzae*. (A) Structures of BadA, Hia and YadA heads with the three domains colored according to the domain annotation from the alignment [15]. The superimpositions of the individual domains from all three proteins are shown in the left panel. Note the different order of domains between Hia and BadA. In the BadA Trp-ring domain, 43 of 45 residues could be superimposed to the equivalent Hia domain with an r.m.s.d. of 2.02 Å, and in the GIN domain, 26 of 30 residues could be superimposed with an r.m.s.d. of 1.58 Å. In the BadA neck region, 19 residues could be superimposed to the YadA neck with an r.m.s.d. of 0.28 Å and to the Hia neck with an r.m.s.d. of 1.32 Å. All r.m.s.d. values refer to the C_α_ atoms. (B) Sequence alignment of the BadA head with other TAAs. The sequences of Hia and YadA are taken from the published structures; alignments based on these structures were used for homology modeling of the BadA head. The conserved residues that were used to name the domains are marked in bold. Abbreviations used: BhBadA – *Bartonella henselae* BadA gi|119890727|, HiHia – *Haemophilus influenzae* Hia gi|21536216|, YeYadA – *Yersinia enterocolitica* YadA gi|28372996|, BqVompD – *Bartonella quintana* VompD gi|49473810|, BbAdh – *Bartonella bacilliformis* adhesin gi|121601790|, SmHypp – *Sinorhizobium meliloti* hypothetical protein gi|15964211|, BmCsup – *Brucella melitensis* cell surface protein gi|17988156|, PcYdlk – *Psychrobacter cryohalolentis* YadA-like protein gi|93006053|, XfSurp – *Xylella fastidiosa* surface protein gi|15838130|.

Comparison of the BadA structure with the two previously determined TAA head structures from YadA [13] and Hia [14] shows that shared domains are structurally nearly identical (Figure 7), even when, as in the case of the Trp-ring and GIN domains, their sequence similarity is barely detectable. In comparing these two domains between BadA and Hia, we find that the structural conservation extends to the conformation of hydrophobic residues in their core. The main differences are two insertions in Hia, one between strands β1 and β2 of the Trp-ring domain and the other between strands β9 and β10 of the GIN domain. Two apparent differences, concerning the relative order of the two domains and the seemingly missing N-terminal strand in Hia GIN, are in fact artifacts of the way the Hia construct was cloned out of the full length gene. Hia contains multiple Trp-ring-GIN-tandems, and the Hia construct was cloned such that the C-terminal GIN domain of one tandem appeared N-terminally to the Trp-ring domain of the next tandem. In the process, the N-terminal strand of the GIN domain, which is detectable in the sequence, was omitted.

All three proteins contain necks, which are structurally nearly identical (Figure 7). The similarity of the necks in BadA and YadA was easily detected at the sequence level. The Hia neck, however, was not recognized before due to sequence divergence, which includes the insertion of a domain of 44 residues (Figure 7). The nearly identical backbone structure in the three necks is the result of a conserved network of mainchain hydrogen bonds and does not seem to involve sidechain interactions, beyond the formation of a small hydrophobic core (Figure 6). The charge network reported in the YadA neck [13] is not present in BadA or Hia and seems to be a specific feature of YadA. In light of these observations, it is hard to understand the exceptional sequence conservation of necks, which is typically in the range of 50% identity between any two necks [4].

### Model of the full BadA head

The part of the BadA head which is not included in our construct shows extensive sequence similarity to the YadA head, allowing us to model it by homology (see Methods). The β-roll domain was modeled using a template-based approach, since YadA contains 8 repeats (whose inner strands carry the conspicuous SVAIG sequence motif) and BadA 11. Two features of BadA could not be modeled for lack of a structural template: (I) the N-terminal segment from the signal sequence cleavage site to the first turn of the β-roll, which is presumably unstructured and also not resolved in the YadA structure, and (II) an insertion in the last turn of the β-roll, which is present in many TAA head domains, but not in YadA [15].

**Figure 8:**
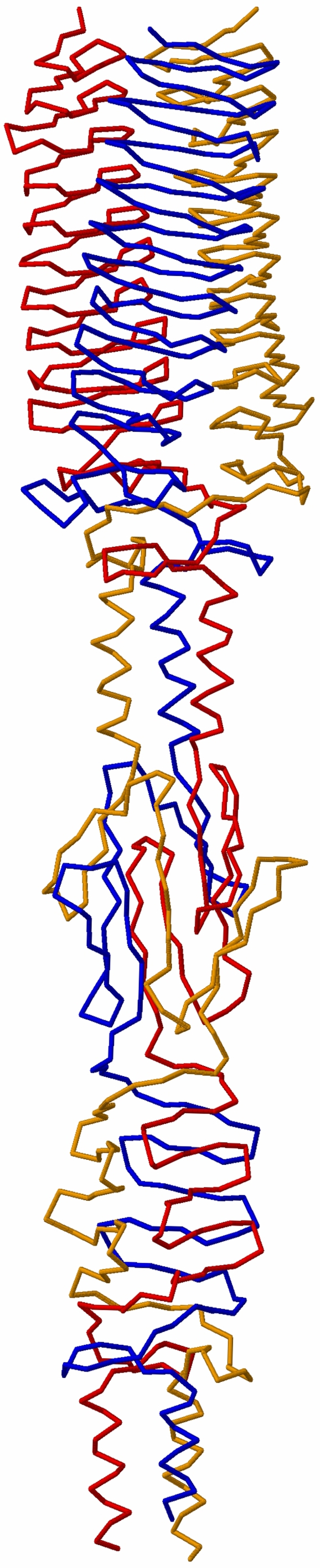
Model of the full BadA head. The head of BadA, comprising the crystal structure of the Trp-ring and GIN domain and models of the YadA-like head and the connecting coiled coil. The structure is heavily intertwined and each chain spirals over 360 degrees around the fiber axis, mostly due to the two neck sequences present.

The similarity between YadA and BadA not only encompasses the left-handed β-roll domain, but also the neck connector and a short coiled-coil segment. Thus, even though the coiled-coil segment N-terminal to the Trp-ring domain was not resolved in our BadA construct, we could merge the model to the structure without gaps by aligning the registers of the coiled coils and modeling the missing part with parametric equations [18]. The resulting model for the complete BadA head is shown in Figure 8.

### Conclusions

We have determined the structure of two domains from the head of the *Bartonella henselae* adhesin BadA. Surprisingly, these domains are structurally nearly identical to two domains from the *Haemophilus* adhesin Hia, despite their similarity being essentially undetectable by sequence comparisons. This is due to the short length of the individual domains, to two large inserts in Hia, and to the seemingly reversed order of the domains in the two proteins. The reversed order is due to the way in which the Hia fragment was constructed; in fact, Trp-ring and GIN domains also occur in tandem in Hia, albeit in multiple copy, and the Hia fragment combines the C-terminal GIN domain of one tandem with the N-terminal Trp-ring domain of the next. The near-identity of the structures is underscored by our ability to solve the BadA structure by molecular replacement, using the Hia structure as a modeling template. From this we conclude that TAA domains retain their structure closely, irrespective of their molecular context, and that their strongly interleaved nature prevents structural divergence, even after considerable sequence divergence has occurred. These findings support our previous proposal that the structure of TAAs can be elucidated by a “dictionary approach”: domains are identified by bioinformatics, compared to a database containing the structures of representative exemplars for each domain, modeled and assembled into complete fibers using the nearly invariant structure of connectors such as the necks and coiled-coil segments [19]. We have laid the bioinformatics groundwork for this approach with a sensitive online system for the annotation of TAA domains [15] and are now in the process of selecting and solving representative exemplars for each domain type.

An important question relates to the role of this part of the BadA head in the adhesive properties of the entire molecule. BadA has been reported to bind to collagen and fibronectin [11], while the homologous Vomps A, B and C of *Bartonella quintana*, which only have the YadA-like part of the head and lack the domains described here, only bind to collagen [20],[21]. For this reason, we suspected that fibronectin binding by BadA would reside in the Trp-ring-GIN tandem. However, attempts to show this remained ambiguous. The fragment binds in isolation to endothelial and epithelial cells and shows a certain affinity for fibronectin in sandwich dotblots, but cannot be co-immunoprecipitated with fibronectin and is insufficient to preserve fibronectin binding in a stalk deletion mutant (Riess, Wagner, Kempf, Ursinus, Linke and Martin, unpublished; Kaiser *et al.*, submitted). We note in this context that the binding affinity of individual heads could be quite low, given their high density on the cell surface. A conspicuous structural feature of the Trp-ring domain are the many open hydrogen bond donor and acceptor groups at the edges of the three β-sheets forming the prism. In the only complex between a bacterial adhesin and fibronectin known to atomic resolution (PDB accession 1o9a; [22]), the interaction is mediated by β-sheet extension along such open edges (“β-zippers”). It is attractive to consider that a similar binding mechanism applies to the Trp-ring domain.

## Materials and Methods

### Protein production and purification

Protein expression and purification of the fragment shown in Figure 2 were performed as described [11]. Note that the fragment was originally considered to be part of the stalk because it showed no homology to the *Yersinia* YadA head [11],[23]. The oligomeric size of the purified protein was verified by gel-sizing chromatography on a calibrated analytical S200 column (GE Healthcare) which was coupled to a MiniDAWN Tristar detector (Wyatt), allowing in addition molecular mass determination by static light scattering.

### Protease resistance and mass spectrometry

For protease resistance assays, the BadA fragment (0.5 mg/ml) was incubated at room temperature in 20 mM MOPS/KOH pH 7.2, 150 mM NaCl with 10 µg/ml of either trypsin or chymotrypsin for 10 min. Reactions were stopped by addition of 1 mM PMSF, and samples were subsequently analyzed by SDS-PAGE and mass spectrometry. In preparation for MS analysis, the proteolytically treated protein was re-purified by ion-exchange and gel-size exclusion chromatography. LC HR MS measurements were performed with an Agilent 1100 series HPLC with a Waters Symmetry C4 3.5 µm column (2.1×100 mm), coupled to a micrOTOFLC mass spectrometer (ESI- TOF, Bruker Daltonics, Bremen, Germany). Protein was eluted from the HPLC column using buffer A (H_2_O/0.05% TFA) and buffer B (CH_3_CN/0.05% TFA) with a gradient from 20–80% buffer B at a flow of 250 µl/min.

### Spectroscopy

Circular dichroism (CD) spectra of proteins (12 µM) were recorded in PBS at 200–240 nm with a J-810 Spectropolarimeter (Jasco), using 1 mm cuvettes. The signal output was converted into molar ellipticity. Thermal stability was monitored by CD spectroscopy using a Peltier-controlled sample holder unit. Temperature profiles at 210 nm were recorded in 1°C increments with 0.2° pitch from 25°C to 100°C. In all cases a temperature probe connected to the cuvette was used to provide an accurate temperature record. The fraction of protein in the unfolded conformation, fU, was calculated as fU = (yF−y)/((yF−yU), where yF and yU represent the values corresponding to folded and unfolded states, respectively, and y being the observed value.

Tryptophan fluorescence was measured at room temperature in PBS buffer at protein concentrations of 30 µM in a FP-6500 spectrofluorometer (Jasco) with λ_ex_ = 293 nm and λ_em_ = 300–400 nm.

### Electron microscopy

Bacterial colonies of BadA^+^ and BadA^−^ strains grown on blood agar [11] were fixed with 2.5% glutaraldehyde in PBS directly on the agar plates for 20 min at ambient temperature and kept for 20 hours at 4°C.

For scanning electron microscopy, colonies were postfixed with 1% osmium tetroxide in 100 mM Phosphate buffer pH 7.2 for 1 h on ice, dehydrated in ethanol and critical-point-dried from CO_2_. The samples were sputter-coated with 8 nm gold-palladium and examined at 20 kV accelerating voltage in a Hitachi S-800 field emission scanning electron microscope.

For transmission electron microscopy, glutaraldehyde-fixed cells were covered with 2% agarose and blocks containing single colonies were cut out. After postfixation with 1% osmium tetroxide in 100 mM Phosphate buffer pH 7.2 for 1 h on ice, these blocks were rinsed with aqua bidest, treated with 1% aqueous uranyl acetate for 1 hr at 4°C, dehydrated through a graded series of ethanol and embedded in Epon. Ultrathin sections were stained with uranyl acetate and lead citrate and viewed in a Philips CM10 electron microscope.

For on-section immunolabeling, cells were fixed with 2.5% glutaraldehyde in PBS, dehydrated in a graded series of ethanol at progressive lower temperature from 0°C down to −40°C, infiltrated with Lowicryl HM20 and UV-polymerized at −40°C. Unspecific binding sites on ultrathin sections were blocked with 0.5% bovine serum albumin and 0.2% gelatine in PBS. Ultrathin sections were then incubated with a BadA specific rabbit IgG antibody (10 µg/ml; raised against the C-terminal part of the BadA head [11]) followed by protein A-10 nm gold conjugates (gift from Dr. Y. Stierhof, Tübingen). Sections were stained with 1% aqueous uranyl acetate and lead citrate and analysed in a Philips CM10 electron microscope at 60 kV using a 30 µm objective aperture.

### Crystallization and data collection

Crystals of the BadA head fragment were obtained at 291 K by the vapor diffusion hanging drop method against one ml of a reservoir solution. Crystal drops were prepared by mixing 1 µl of protein at 11 mg/ml concentration with 1 µl of reservoir solution. Crystals were obtained with 0.05 M ammonium sulfate, 0.05 Bis-Tris pH 6.5, 30% v/v pentaerythrol ethoxylate with a size of 150×100×100 µm. Single crystals were flash-frozen in their mother liquid and data collection was performed at 100 K. The crystal system is triclinic P1 with cell constants of a = 29.87 Å, b = 51.140 Å, c = 58.62 Å – α = 65.87, β = 76.60, γ = 82.08. The crystals contained one trimer in the asymmetric unit, diffracted to a resolution limit of 1.13 Å and showed a solvent content of 41%. A high and low resolution data set was collected at beamline ID29, ESRF (European synchrotron radiation facility). Data were indexed, integrated and scaled with the XDS program package [24]. High and low resolution data were merged using the XSCALE subroutine of the XDS package.

### Bioinformatics

The homology of the N-terminal part of the BadA head with YadA was found using PSI-BLAST [25] (E-value of 5e-07 in the first iteration). Searches for distant homologs of known structure to BadA were performed with three standard programs that use sequence-profile comparisons (PSI-BLAST), sequence-HMM comparisons (SAM-T02 [26]), and profile-profile comparisons (COMPASS [27]). In addition, we used a structure prediction metaserver (3D-Jury [28]). None of these tools yielded significant matches. More recently we developed a tool based on HMM-HMM comparisons, which was shown to be at least twice as sensitive in detecting distant homologs as the methods listed above; this tool, HHsearch [29], was implemented in a web server, HHpred [30]. The homology between Hia and BadA was detected using the HHPred server, running HHsearch 1.1.4. in its default settings, albeit with low statistical significance (E-values of 0.93 and 3.9 and probability of 66% and 30% for the Trp-ring and GIN domains, respectively). Note that with the current HHsearch version 1.5.0, HHpred returns good statistical significances (probabilities of 80–90%) for the matches between BadA and Hia, but only if the compositional bias correction is turned off in the ‘more options’ field.

Sequences of corresponding domains were manually aligned with respect to secondary structure arrangement and conserved residues. Homology models based on the structures of YadA and Hia were built with the nest program from the Jackal package (http://wiki.c2b2.columbia.edu/honiglab_public/index.php/Software:Jackal). Each chain was modeled separately, then all chains were combined together and sterical clashes were removed with the profix program, again from the Jackal package.

The YadA-like domain of the BadA head was modeled on a template structure containing three partially overlapping core sections from the YadA structure and a following neck sequence. Preparing this template was necessary, as this domain in BadA is significantly longer than in YadA; it has 11 head repeats instead of 8. Moreover, we had to introduce a break in the last repeat before the neck, since BadA has a conserved insertion in that place (data not shown) for which we do not have a structural template.

The coiled-coil segment preceding the Trp-ring domain has a periodicity of 11 and was constructed with BeammotifCC [18]. Its transition from the neck into the Trp-ring domain was modeled based on the structures of YadA and Hia.

The model of the full head of BadA was constructed using the solved structure described here and the two models mentioned above. The necessary structural superimpositions were done with VMD [31].

### Structure determination and refinement

The structure of the BadA head fragment was solved by molecular replacement using models based on the PDB coordinates of the partial head of *Haemophilus* Hia (1s7m). Two subdomains of this model were independently placed using the program MOLREP [32] and initially refined in REFMAC [33]. To improve this model, the program packages ARP/wARP [34], Coot [35], and REFMAC were used to rebuild sidechains and to add missing residues. A random set of 5% of the data were neglected during the refinement process and marked as test set for cross-validation. Atoms were refined anisotropically and TLS parameters for the three independent protein chains were defined using REFMAC [36]. ARP/wARP was used to build the solvent structure. Together, this procedure returned a final model consisting of 2870 non-hydrogen atoms and 438 water molecules (corresponding to residues 385–498 in chains A and C and to residues 385–495 in chain B). Together with the hydrogen atoms generated for all amino acid residues, a crystallographic R/R_free_-factor of 0.156/0.184 was achieved. Model superposition was performed by the programs top3d or LSQ included in the CCP4 program package [37]. Secondary structure elements were defined according to DSSP criteria (http://molbio.info.nih.gov/structbio/basic.html). Figures were prepared using the programs DINO (http://www.dino3d.org/) and Rasmol (http://www.openrasmol.org/).

### Coordinates

The x-ray structure was deposited in the Protein Data Bank (PDB, access code 3D9X). The model of the full BadA head can be downloaded from http://protevo.eb.tuebingen.mpg.de/coordinates/.

## Supporting Information

Figure S1 Mass Spectrometry Analysis, Supplement to Figure 2
(2.60 MB PDF)
