# Supplementary material for: Structure of the Head of the Bartonella Adhesin BadA
Source: PLoS Pathog. 2008 Aug 8;4(8):e1000119. doi: 10.1371/journal.ppat.1000119 (PMC2483945; doi:10.1371/journal.ppat.1000119)

BadA head fragment sequence

MKALRGMISEKGGWNLTVNNDNNTVVSSGGALDLSSGSKNLKIVKDGKKNNVT
FDVARDLTLKSIKLDGVTLNETGLFIANGPQITASGINAGSQKITGVAEGTDANDA
VNFGQLKKIETEVKEQVAASGFVKQDSDTKYLTIGKDTDGDTINIANNKSDKRT

Trypsin fragments found:

GMISEKGGWNLTVNNDNNTVVSSGGALDLSSGSKNLKIVKDGKKNNVTFDVAR
DLTLKSIKLDGVTLNETGLFIANGPQITASGINAGSQKITGVAEGTDANDAVNFG
QLKKIETEVKEQVAASGFVKQDSDTK

13941 Da

GMISEKGGWNLTVNNDNNTVVSSGGALDLSSGSKNLKIVKDGKKNNVTFDVAR
DLTLKSIKLDGVTLNETGLFIANGPQITASGINAGSQKITGVAEGTDANDAVNFG
QLKKIETEVKEQVAASGFVK

13266 Da

Chymotrypsin fragments found:

RGMISEKGGWNLTVNNDNNTVVSSGGALDLSSGSKNLKIVKDGKKNNVT
FDVARDLTLKSIKLDGVTLNETGLFIANGPQITASGINAGSQKITGVAEGTD
ANDAVNFGQLKKIETEVKEQVAASGFVKQDSDTKYL

14374 Da

RGMISEKGGWNLTVNNDNNTVVSSGGALDLSSGSKNLKIVKDGKKNNVT
FDVARDLTLKSIKLDGVTLNETGLFIANGPQITASGINAGSQKITGVAEGTD
ANDAVNFGQLKKIETEVKEQVAASGFVKQDSDTKY

14261 Da

RGMISEKGGWNLTVNNDNNTVVSSGGALDLSSGSKNLKIVKDGKKNNVT
FDVARDLTLKSIKLDGVTLNETGLFIANGPQITASGINAGSQKITGVAEGTD
ANDAVNFGQLKKIETEVKEQVAASGF

13195 Da

**Analysis Info**	**Trypsin digest, 8.9-9.1 min**	Acquisition Date	12/13/2006 6:27:58 PM
Analysis Name	D:\Data\12_2006\MT0949_Albrecht_BADA6-T_3_01_1531.d		
Method	lc600-3000.m	Operator	Administrator
Sample Name	MT0949_Albrecht_BADA6-T	Instrument	micrOTOF
Comment			

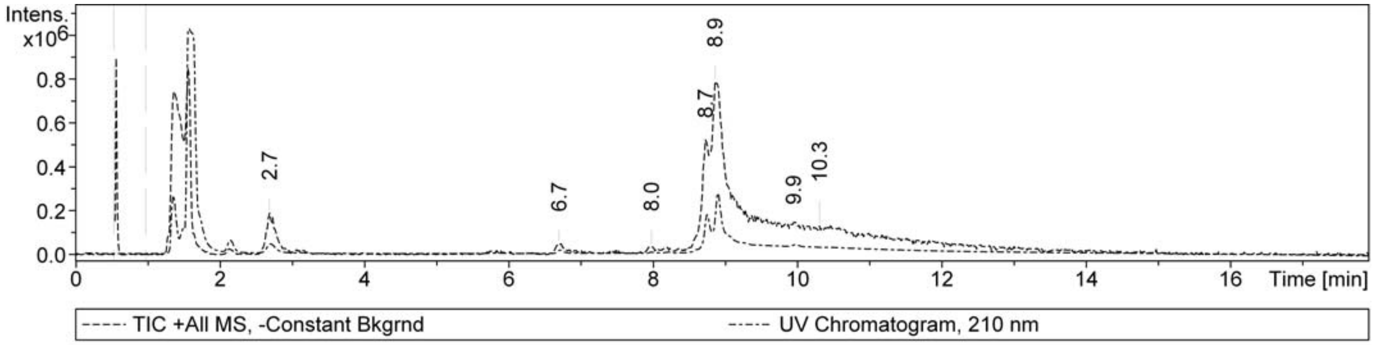

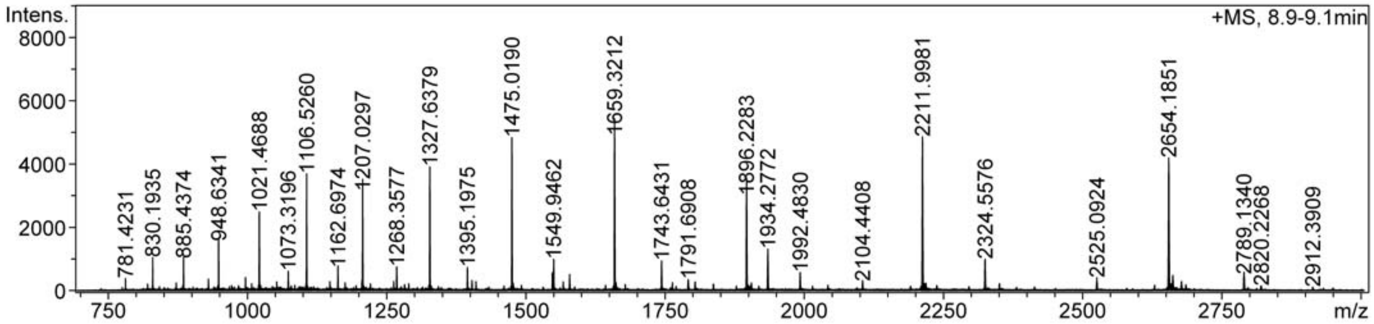

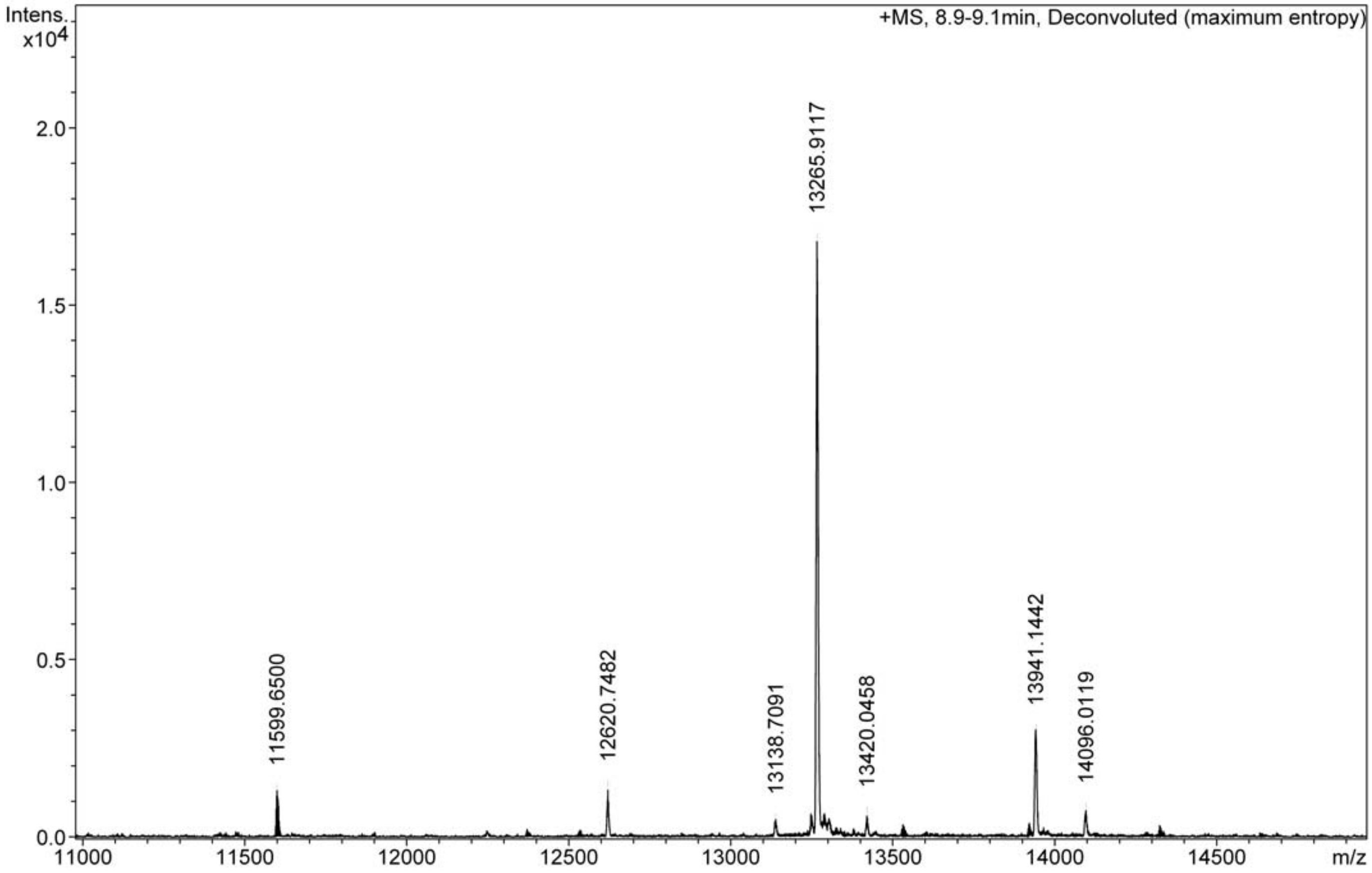

**Analysis Info**	**Trypsin digest, 10.4-10.7 min**	Acquisition Date	12/13/2006 6:27:58 PM
Analysis Name	D:\Data\12_2006\MT0949_Albrecht_BADA6-T_3_01_1531.d		
Method	lc600-3000.m	Operator	Administrator
Sample Name	MT0949_Albrecht_BADA6-T	Instrument	micrOTOF
Comment			

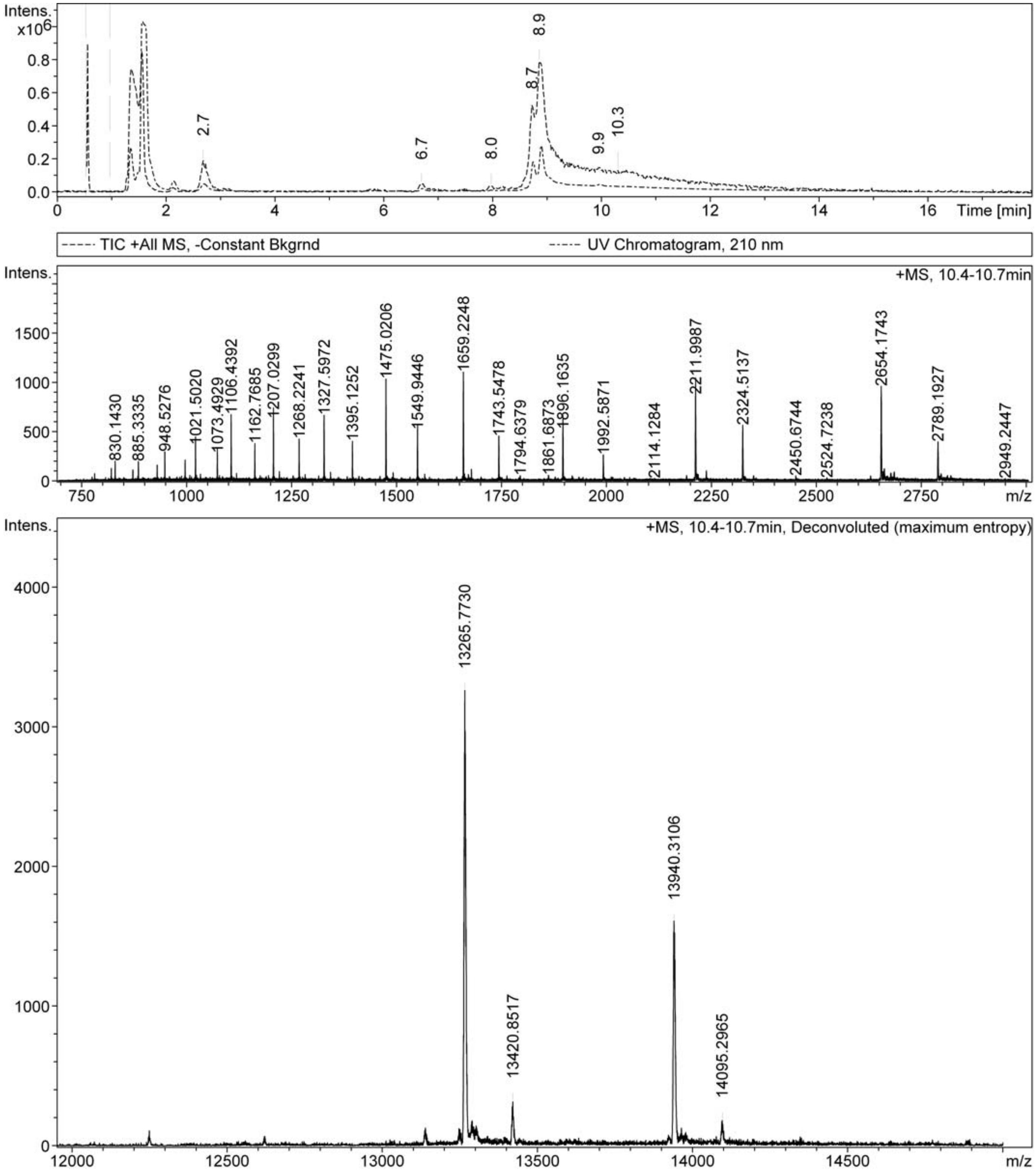

**Analysis Info**	**Chymotrypsin digest, 9.0-9.3 min**	Acquisition Date	12/13/2006 7:00:33 PM
Analysis Name	D:\Data\12_2006\MT0950_Albrecht_BADA6-CT_4_01_1532.d		
Method	lc600-3000.m	Operator	Administrator
Sample Name	MT0950_Albrecht_BADA6-CT	Instrument	micrOTOF
Comment			

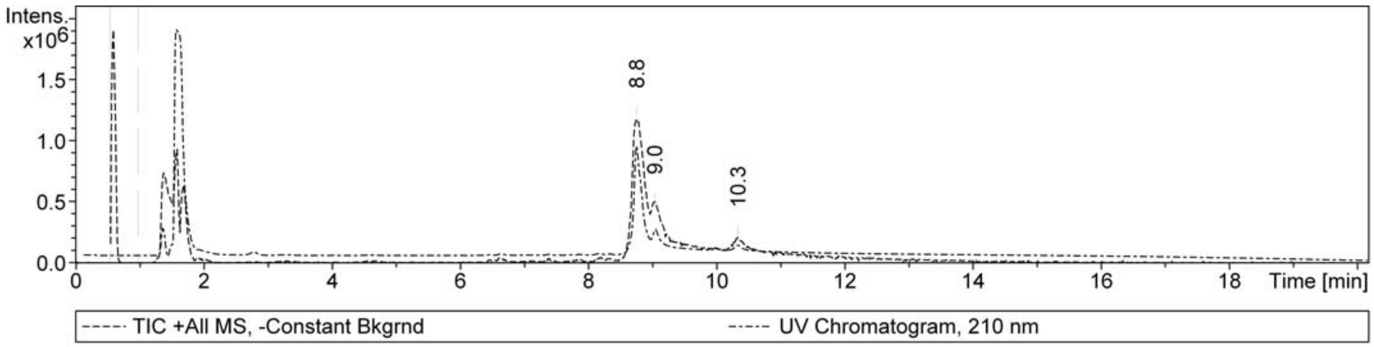

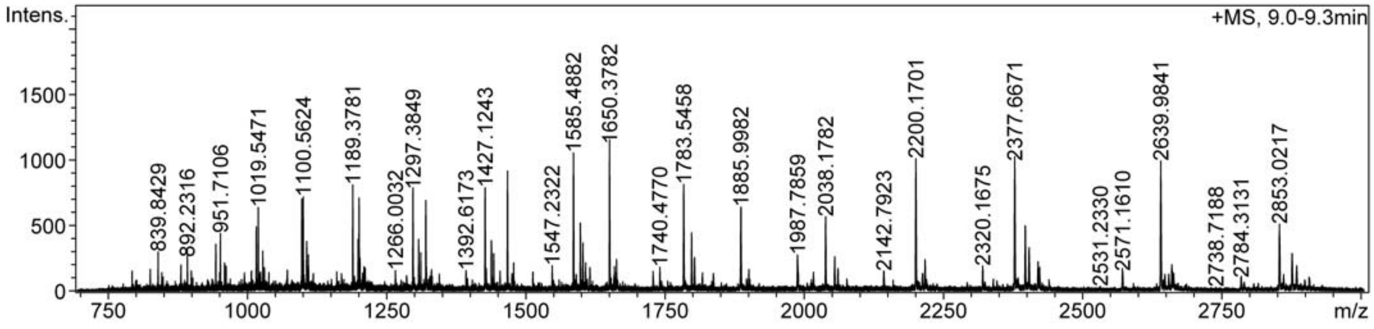

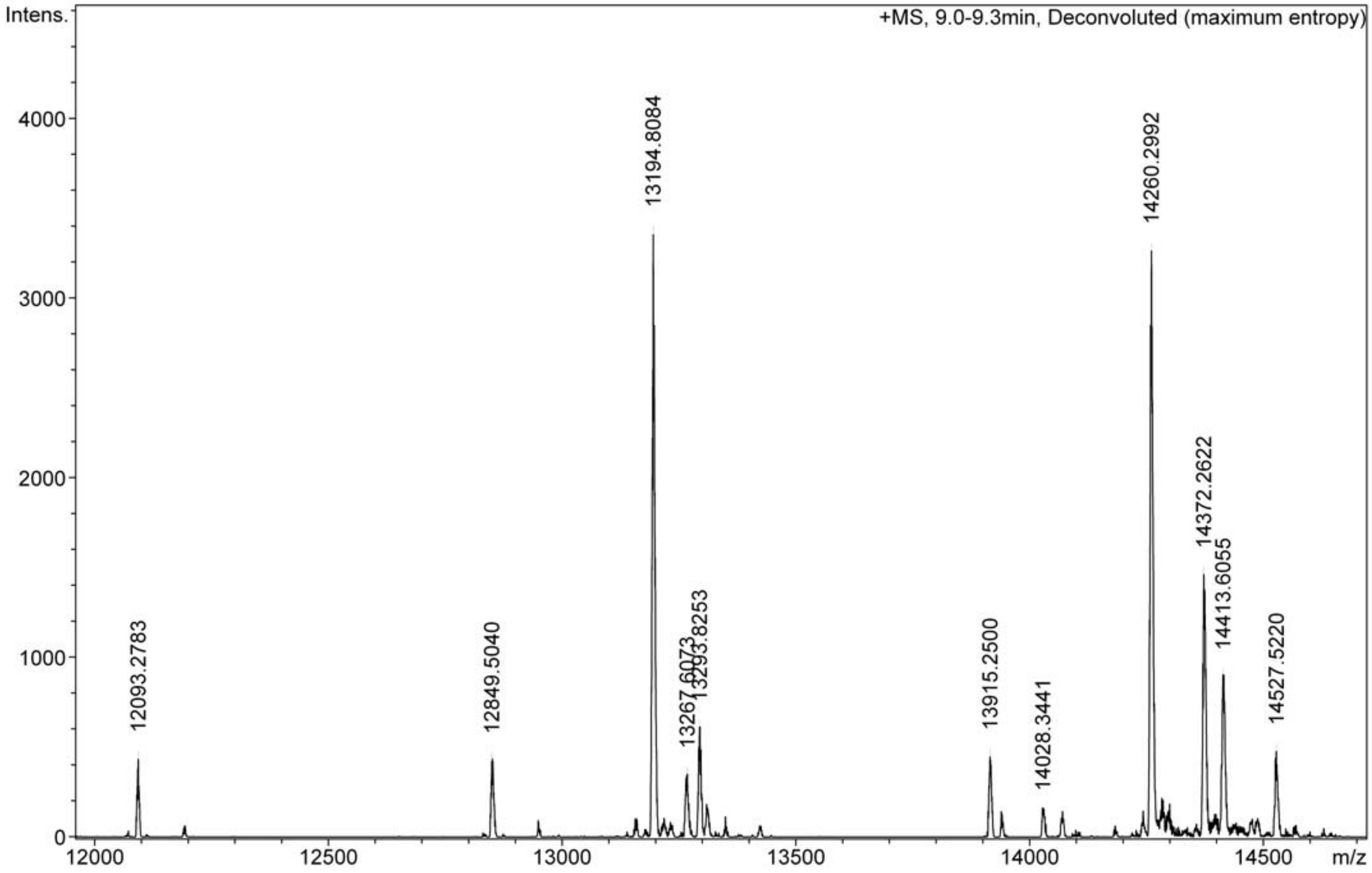

**Analysis Info**	**Chymotrypsin digest, 8.6-8.8 min**	Acquisition Date	12/13/2006 7:00:33 PM
Analysis Name	D:\Data\12_2006\MT0950_Albrecht_BADA6-CT_4_01_1532.d		
Method	lc600-3000.m	Operator	Administrator
Sample Name	MT0950_Albrecht_BADA6-CT	Instrument	micrOTOF
Comment			

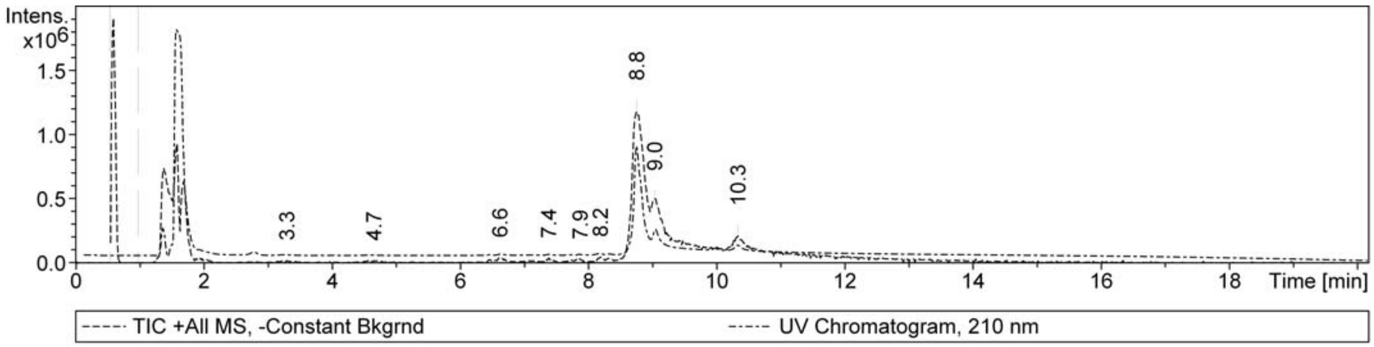

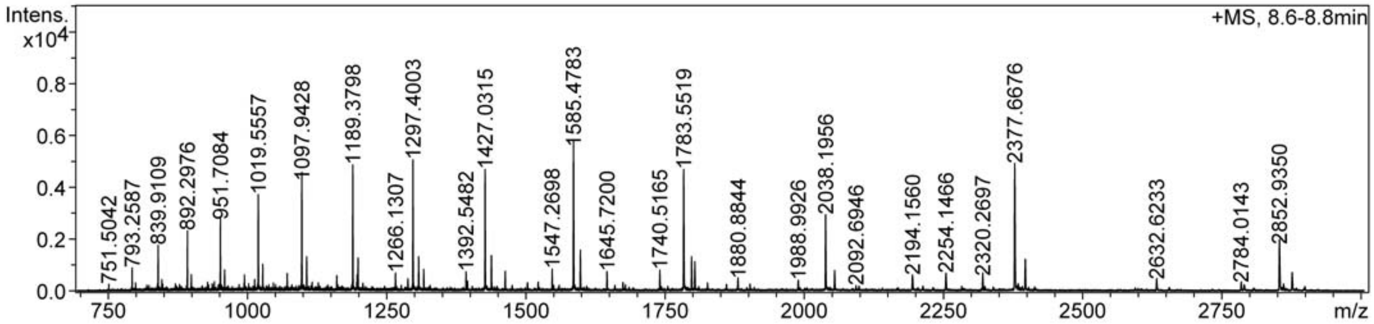

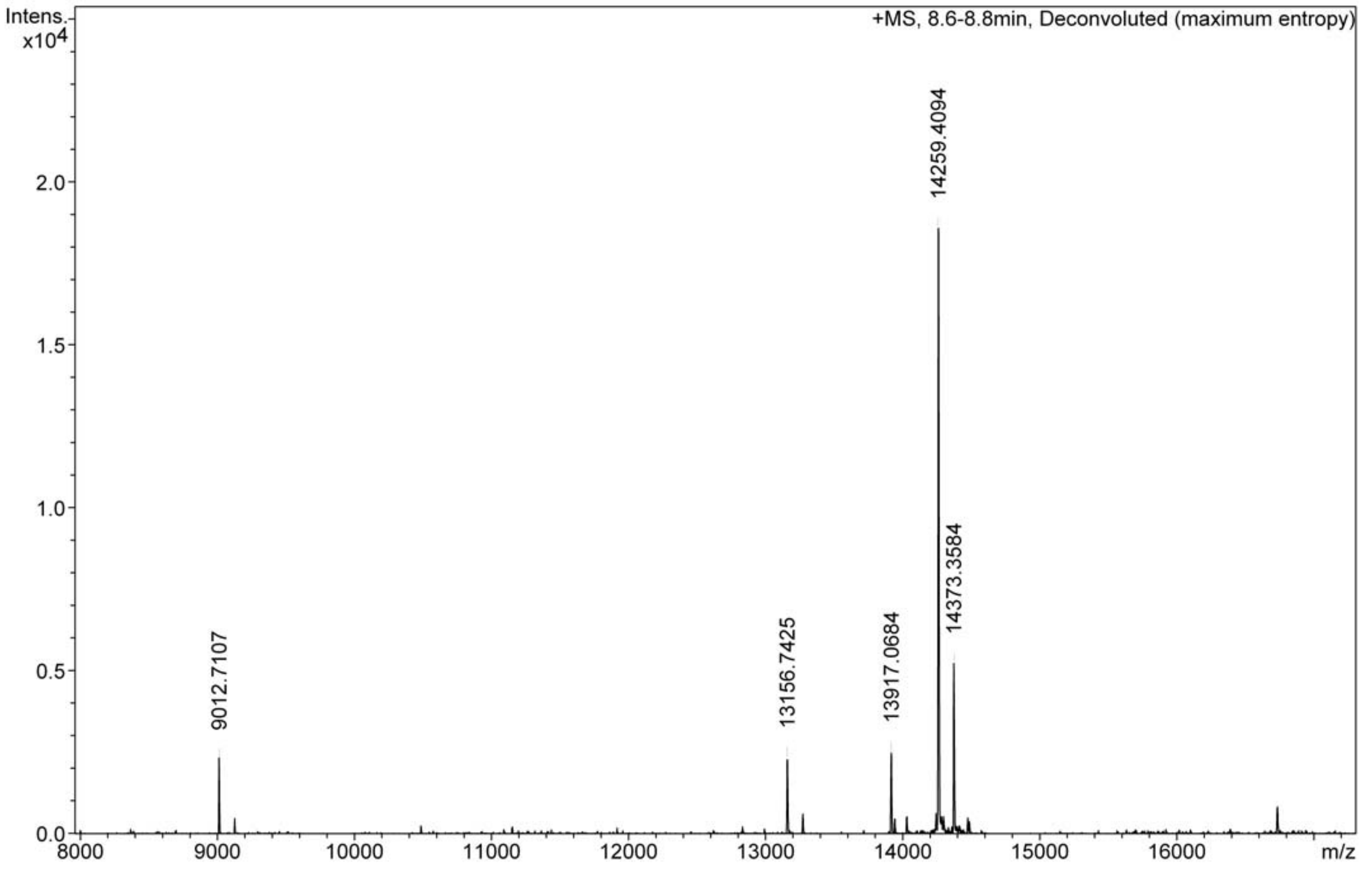

**Analysis Info**	**Chymotrypsin digest, 10.2-10.4 min**	Acquisition Date	12/13/2006 7:00:33 PM
Analysis Name	D:\Data\12_2006\MT0950_Albrecht_BADA6-CT_4_01_1532.d		
Method	lc600-3000.m	Operator	Administrator
Sample Name	MT0950_Albrecht_BADA6-CT	Instrument	micrOTOF
Comment			

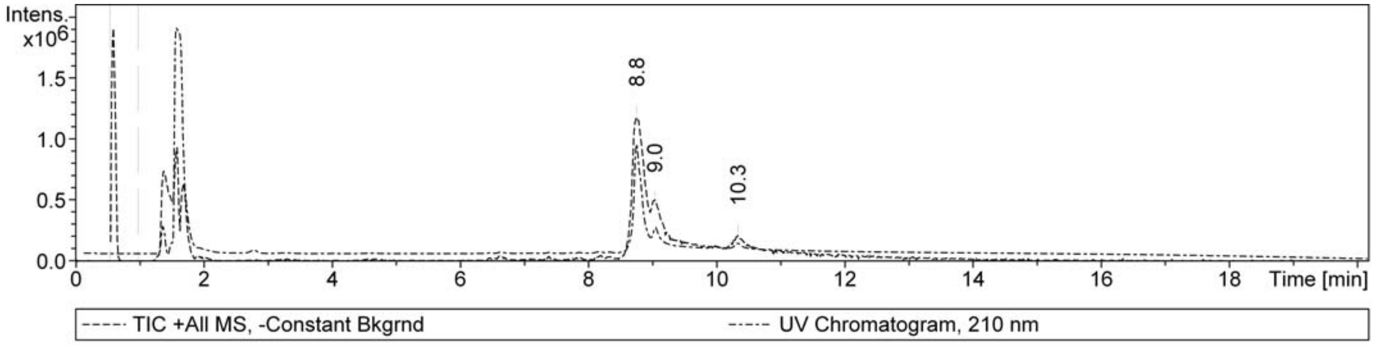

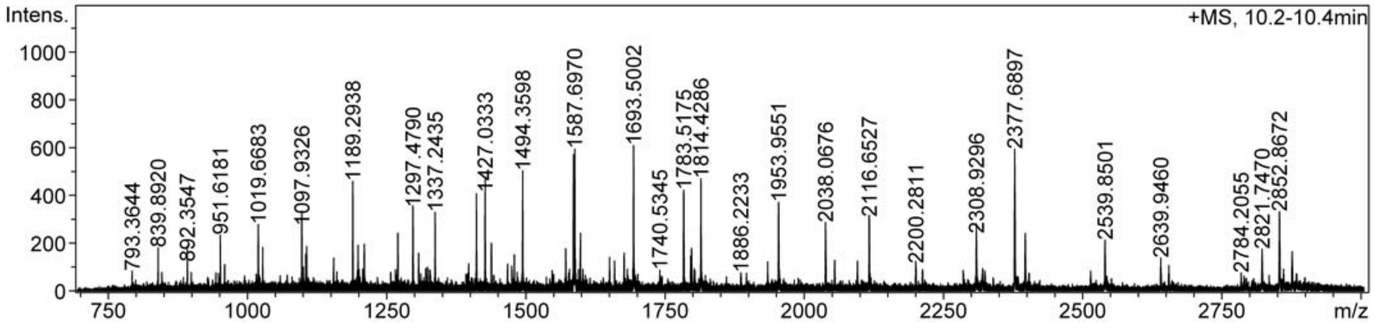

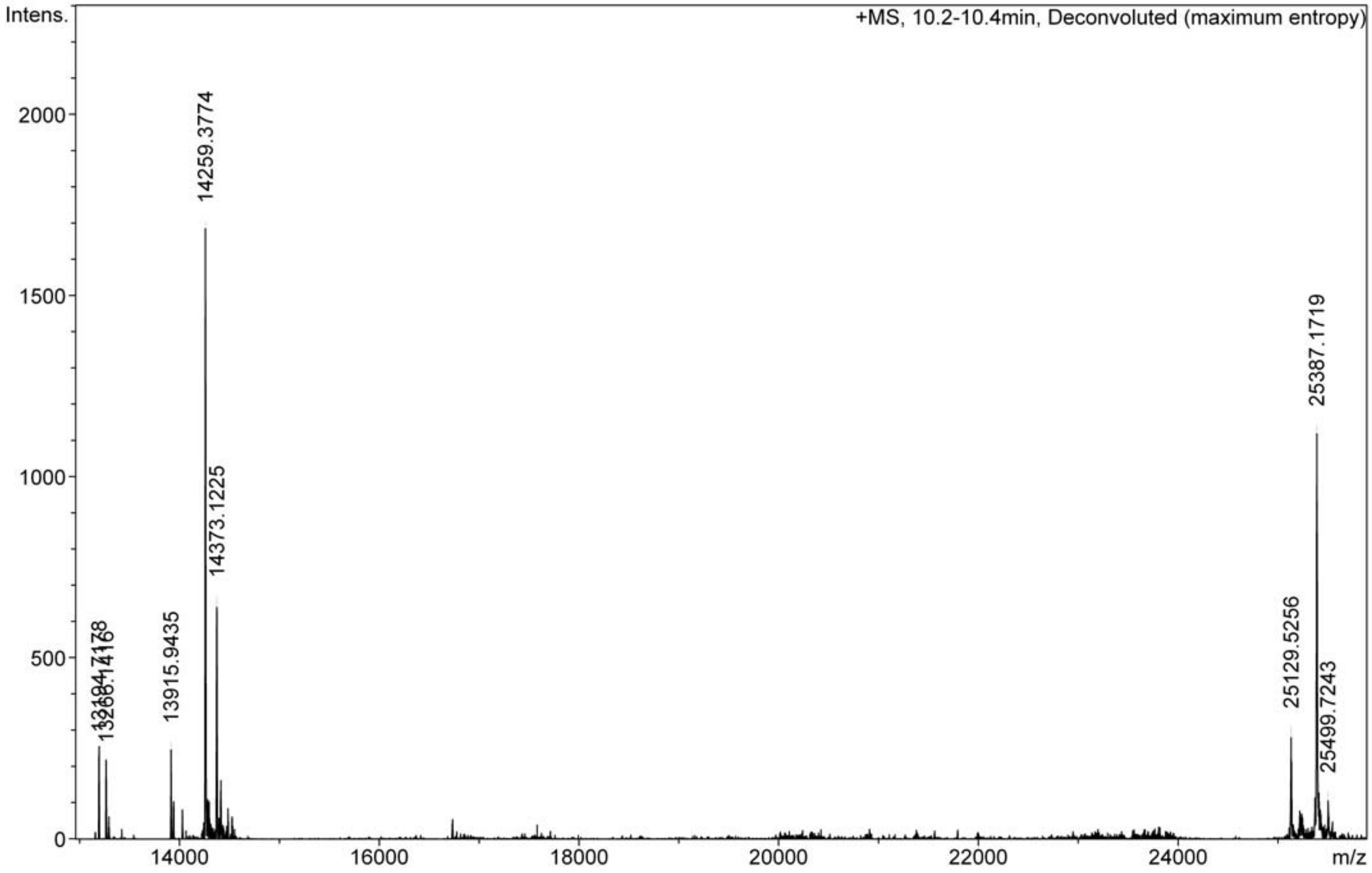

Supplement: Figure S1 — Mass Spectrometry Analysis, Supplement to Figure 2 (2.60 MB PDF) [file ppat.1000119.s001.pdf]
